# Object detection based on an adaptive attention mechanism

**DOI:** 10.1038/s41598-020-67529-x

**Published:** 2020-07-09

**Authors:** Wei Li, Kai Liu, Lizhe Zhang, Fei Cheng

**Affiliations:** School of Computer Science and Technology, Xidian University, Xi’an, 710071 China

**Keywords:** Computational science, Computer science, Information theory and computation, Information technology, Electrical and electronic engineering

## Abstract

Object detection is an important component of computer vision. Most of the recent successful object detection methods are based on convolutional neural networks (CNNs). To improve the performance of these networks, researchers have designed many different architectures. They found that the CNN performance benefits from carefully increasing the depth and width of their structures with respect to the spatial dimension. Some researchers have exploited the cardinality dimension. Others have found that skip and dense connections were also of benefit to performance. Recently, attention mechanisms on the channel dimension have gained popularity with researchers. Global average pooling is used in SENet to generate the input feature vector of the channel-wise attention unit. In this work, we argue that channel-wise attention can benefit from both global average pooling and global max pooling. We designed three novel attention units, namely, an adaptive channel-wise attention unit, an adaptive spatial-wise attention unit and an adaptive domain attention unit, to improve the performance of a CNN. Instead of concatenating the output of the two attention vectors generated by the two channel-wise attention sub-units, we weight the two attention vectors based on the output data of the two channel-wise attention sub-units. We integrated the proposed mechanism with the YOLOv3 and MobileNetv2 framework and tested the proposed network on the KITTI and Pascal VOC datasets. The experimental results show that YOLOv3 with the proposed attention mechanism outperforms the original YOLOv3 by mAP values of 2.9 and 1.2% on the KITTI and Pascal VOC datasets, respectively. MobileNetv2 with the proposed attention mechanism outperforms the original MobileNetv2 by a mAP value of 1.7% on the Pascal VOC dataset.

## Introduction

Object detection is a basic task in computer vision. Many other computer vision tasks, such as object tracking and image segmentation, can benefit from fast and accurate object detection results. Over the past few decades, many object detection methods have been proposed. Early methods mainly deployed handcrafted features and shallow machine learning models. These methods were vulnerable to overfitting and often included a large amount of calculations. Recently, convolutional neural networks have been used to learn feature representations from images automatically and are the dominant approach for object detection.

Convolutional neural networks (CNNs) progressively extract semantic information from input images through convolution layers and discards pixels on the feature maps that are less informative, forcing the attention of the CNNs to focus on the more informative pixels within the spatial dimension.

Learning from previous works, the performance of CNNs can be boosted through strengthening the representative ability of different dimensions. VGG^1^ and ResNet^2^ have more layers than previous CNNs such as AlexNet^3^. They encode higher-dimensional features so that the classifier can differentiate positive and negative samples more easily. DenseNet^4^ uses dense connections to integrate information from different layers which also boosts the performance. ResNeXt^5^ and Xception^6^ also lift the performance effectively by expanding the cardinality dimension. Some works^7–9^ exploit an attention mechanism on the channel dimension or/and spatial dimension to improve the performance of CNNs.

Apart from the design schemes of CNNs mentioned above, we aim to improve their representative ability by strengthening other dimensions, namely, channel-wise attention, spatial-wise attention and domain attention in a fully data-driven manner. Channel-wise attention performs feature recalibration with respect to the channel dimension. On the one hand, we obtain channel-wise attention tensors from both global max pooling and global average pooling the input feature maps. On the other hand, we obtain spatial-wise attention tensors from fully convolutional layers. Because the proposed attention units are fully data drived, we call them adaptive attention units. Through adaptive attention, a CNN learns to use global and local information to selectively emphasize informative features and suppress less useful ones.

Previous works^10,11^ have proposed ’adaptive attention’. Lu et al.^10^ proposed a novel adaptive attention model (AAM) with a visual sentinel for image captioning. Recent neural models for image captioning usually employ an encoder–decoder framework with an attention mechanism. In such frameworks, a CNN-based image encoder is used to extract feature vectors for an input image, and an RNN-based caption decoder is used to generate caption words recurrently. In contrast to the attention based encoder–decoder framework that relies on only a visual signal or the a language model, the AAM can automatically decide when to look at an image (visual signal) and when to rely on the language model. Based on the AAM, their methods significantly outperform other state-of-the-art approaches on COCO and Flickr30k. Lun Huang^11^ etc. proposed an attention model, namely, adaptive attention time (AAT), to automatically align the source and the target for image captioning. As opposed to the attention mechanism that assumes the one-to-one or one-to-fixed-number of steps to map from source image regions and target caption words, which is never possible, AAT allows the framework to learn how many attention steps to take to output a caption word at each decoding step (one-to-many mapping). Experiments also show that the proposed AAT outperforms previously published image captioning models. In the current paper, we proposed an adaptive domain attention unit. It automatically weights the two kinds of channel-wise attentions generated by the two-branch structure without predefined parameters. Because the domain attention generated by the domain attention unit and the output channel attention only depend on the features of the input data, we call the output of the proposed module the adaptive channel-wise attention.

YOLOv3^12^ is a kind of CNN with a high inference speed and detection accuracy performance that uses DarkNet53 as a backbone network. It contains many $1\times 1$ kernels to extract important information and strengthen the nonlinear representation ability. It also utilizes residual connections and multi-scale detectors that greatly improve its performance. MobileNetv2^13^ is built upon depth inverted residual modules(IRMs). Each IRM contains depth-separable convolution, point-wise convolution, and an inverted residual connection. Detection models built upon MobileNetv2 can run extremely fast. In this work, we test the proposed mechanisms on YOLOv3 and MobileNetv2.

The contributions of our work are as follows:

1. We designed three novel adaptive attention units: an adaptive channel-wise attention unit, an adaptive spatial-wise attention unit and an adaptive domain attention unit.

2. The proposed adaptive attention mechanism is fully data driven, lightweight and easy to apply.

3. We applied an adaptive attention mechanism to YOLOv3 and MobileNetv2. Experiments are performed on the KITTI and Pascal VOC datasets that show that the proposed model achieves a better performance compared to the original YOLOv3 and MobileNetv2 implementation.

## Related works

Many CNNs are applied to object detection tasks. These CNNs can be roughly divided into two-stage methods and single-stage methods. Two-stage methods consist of a stage for region proposal generation and another stage for positive sample classification and localization. Single-stage methods treat the background as the $(c+1)$th class (*c* is the number of positive classes) and resolve the object detection task as a regression problem. Single-stage methods outperform two-stage methods in terms of inference speed by a large margin.

Ross et al. proposed the R-CNN^14^. The R-CNN divides the object detection task into two stages. In the first stage, a selective search method is used to generate thousands of region proposals. In the second stage, the classification task and bounding box regression task on these region proposals are finished simultaneously. The R-CNN is the first work that uses a CNN to solve the object detection task. Despite the pioneering aspects of R-CNNs, they are time consuming because they process each region proposal independently. The Fast R-CNN^15^ remedies this by shared convolution computing. It processes each input image as a whole and obtains the feature maps of each input. ROI pooling is used to obtain the same sized feature maps of each proposal so that they can fit into subsequent fully connected layers. Running Fast R-CNN is 9 times and 213 times faster than R-CNN during training and testing stages, respectively. Faster R-CNN^16^ improves the the performance of Fast R-CNN by replacing the selective search module with a region proposal network (RPN). The RPN generates anchor boxes of different sizes and aspect ratios. They provide the initial position and scale of the predicted bounding boxes. The RPN outputs the regressed anchor boxes and a tag indicating if it is a positive sample. These outputs are fed into subsequent networks that act as a multi-class classifier and a bounding box regressor. Faster R-CNN can be trained end-to-end and has a better performance than its predecessors. Many two-stage methods have been proposed after Faster R-CNN, such as MS-CNN^17^, cascading R-CNN^18^, and so on. Many of them are based on the aforementioned models.

SSD^19^, YOLOv1^20^ and their derived versions are the representative works of single-stage detectors. SSD is a fully convolutional network. VGG16 is applied as its backbone network for feature extraction, and it deploys multi-scale features for object detection. It can be trained end-to-end and had a great impact on the succeeding works. YOLOv1 divides the input images into $7\times 7$ grades. If the centre of an object falls into one of the grades, that grade is responsible for the detection of the object. This means that if the centre of two objects falls into the same grade, only one object can be correctly detected. Furthermore, the last two layers of YOLOv1 are fully connected layers. As a result, the inputs of YOLOv1 should be resized to the same scale, making YOLOv1 less flexible. YOLOv2^21^ remedies these defects through constructing a fully convolutional network and introducing the anchor mechanism. YOLOv2 also exploit the K-means algorithm to select initial sizes and aspect ratios of anchor boxes. While it is still fast, the detection precision and performance on small object detection remain to be improved. YOLOv3 integrated many advantageous design concepts of the CNN, such as residual connections, $1\times 1$ convolution kernels, and detectors with multi-scale features which balance the performance between precision and speed. Many single-stage methods have been proposed after SSD, YOLO and their derived versions, such as DSSD, RSSD, Tiny YOLO with improved performance in accuracy or inference speed. MobileNets^13,22,23^ run extremely fast with a slight accuracy decrease. Apart from these good design concepts, we improve YOLOv3 through a novel fully data-driven attention mechanism.

Jie et al. proposed squeeze-and-excitation module (SE module)^7^. It is a light plug-in module that allows the network to perform feature recalibration through which the network learns to use global information to selectively emphasize informative features and suppress less useful ones. In their work, global information is obtained by a global average pooling operation. Sanghyun et al. proposed the convolutional block attention module (CBAM)^8^. They gather global information through both global average pooling and global max pooling because global max pooling gathers finer channel-wise attention. Moreover, they also devised a spatial attention module through an inter-spatial relationship of features. Different from channel-wise attention that focuses on ‘what’ to attend to, the spatial attention focuses on ‘where’ as an informative part. CBAM gathers channel-wise attention and spatial-wise attention in a sequential manner. Jongchan et al. also exploited both channel and spatial attention and proposed the bottleneck attention module (BAM)^9^. In their work, BAM gathers channel-wise attention and spatial-wise attention in a parallel manner.

Despite the good design concepts of^10,11^, they weight global average pooling and global max pooling equally. Given an input feature map, global average pooling tends to identify discriminative regions of an object, while small object can be more beneficial from global max pooling that identifies global max values. Given a set of input images, the distribution of objects size may not be uniform; thus, equally weighting the global average pooling and global max pooling may have a negative impact on the detection performance of objects, both large and small. Based on the above analysis, three novel and fully data-driven attention units, namely, a channel-wise attention unit, a domain attention unit, and a space-wise attention unit, are proposed. The proposed domain attention unit adaptively weights the two attention tensors obtained by the adaptive channel-wise attention units. For adaptive space-wise attention, because lower layers of a network contain abundant positional information but less semantic information, and higher layers contain abundant semantic information but less positional information, we only apply spatial attention to several lower layers of a detection network and apply channel attention to several higher layers of a detection network. The key novelty of our methods lies in the domain attention unit. Notably, our domain attention is different from other models with the same name. Our domain attention inherits the merits of both global average pooling and global max pooling. The inputs of our domain attention unit are the outputs of the two sibling squeeze-and-excitation modules. The domain attention in^24^ is used for domain adaptation and consists of a fully connected layer and a nonlinear layer. Its input is the feature vector produced by a global average pooling layer. The feature vector is also fed into the SE unit in their work.

## Method description

**Figure 1:**
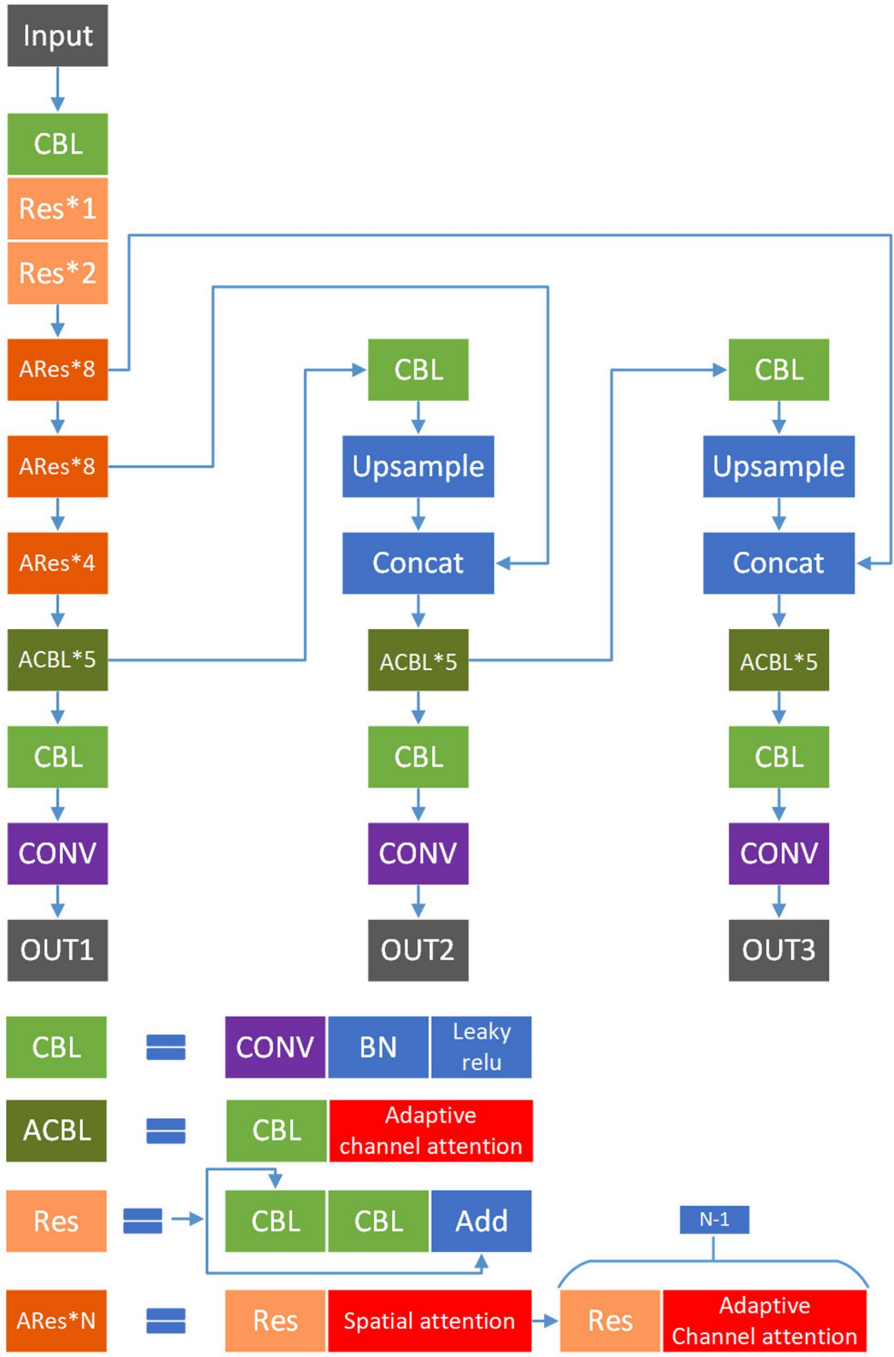
Flowchart of YOLOv3 architecture with adaptive attention.

**Figure 2:**
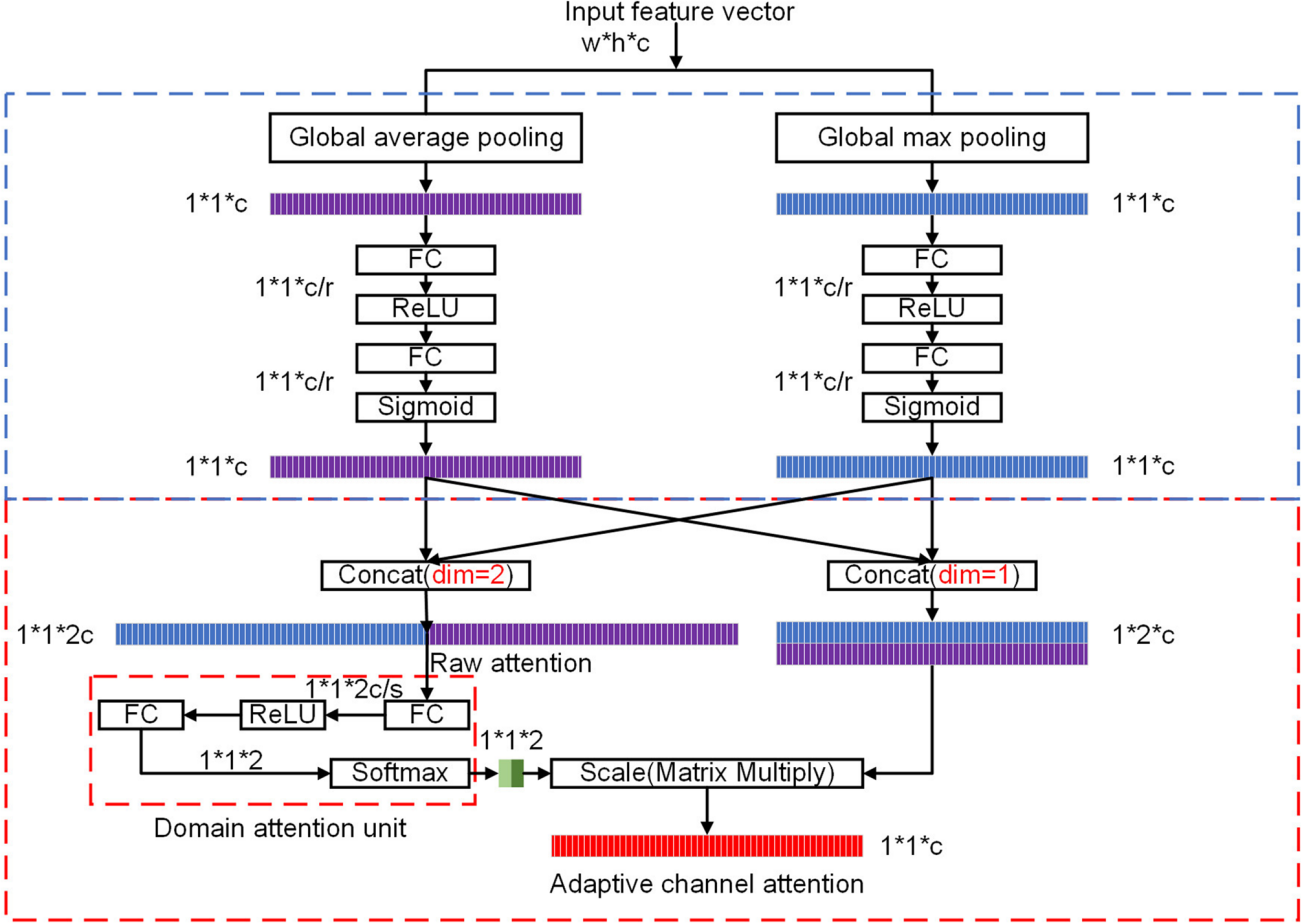
Adaptive channel-wise attention units. r and s are compression ratios.

In this section, first, the proposed network structure is introduced. Then, the adaptive channel-wise attention, adaptive domain attention and spatial-wise attention are described. As shown in Fig. 1, the above modules are integrated into YOLOv3. Spatial attention modules reside after the first ‘Res’ block in ‘ARes*N’ blocks as these lower layers contain abundant positional information but less semantic information. Channel attention units and domain attention units separately reside after the remaining seven ‘Res’ blocks. Channel attention units and domain attention units also reside in each ‘ACBL’ module after each ‘CBL’ module as these higher layers contain more semantic information but less positional information. Note that domain attention units reside in channel attention units. The detailed structure of the adaptive channel attention units is shown in Fig. 2.

### Adaptive channel-wise attention

We obtain adaptive channel-wise attention by the squeeze-and-excitation structure and a domain attention unit that acts as a calibrator of the outputs of the adaptive channel-wise attention units. Different from SENet, which only considered global average pooling when designing SEBlock, we consider that both global average pooling and global max pooling are useful. The basic intuition behind this is that given an input feature map, global average pooling tends to identify the object extent. On the other hand, the global max point identified by global max pooling indicates that the position contains the feature of an object that can be used for the detection task. Global max pooling is more useful when the object is small and when the scale of feature map shrinks considerably with respect to the spatial dimension during forward propagation.

Although several works have used both global average pooling and global max pooling for channel-wise attention, they weight the two kinds of attentions equally. In some cases, that is sub-optimal because the two kinds of attentions emphasize different aspects of a feature map. For example, the KITTI dataset contains many objects of various sizes. Weighting the two kinds of attentions equally may have a negative impact on other objects.

The key novelty of our methods lies in the domain attention unit. For designing the domain attention unit, several preconditions need to be met. 1. It should be fully data driven. Its intermediate values and output can adapt to the input data. 2. It is sufficiently powerful to weight raw attention vectors. 3. It should be as light as possible to minimize the computational overhead. As a result, it is natural to consider feature-based attention mechanisms for weighting the raw attention tensors. Furthermore, the SE module that accounts for the channel-wise attention is constructed by fully connected layers with only one hidden layer. Other works have also proven its effectiveness and efficiency^10,11^. Hence, we use a simple method to construct the domain attention module that consists of three fully connected layers.

**Figure 3:**
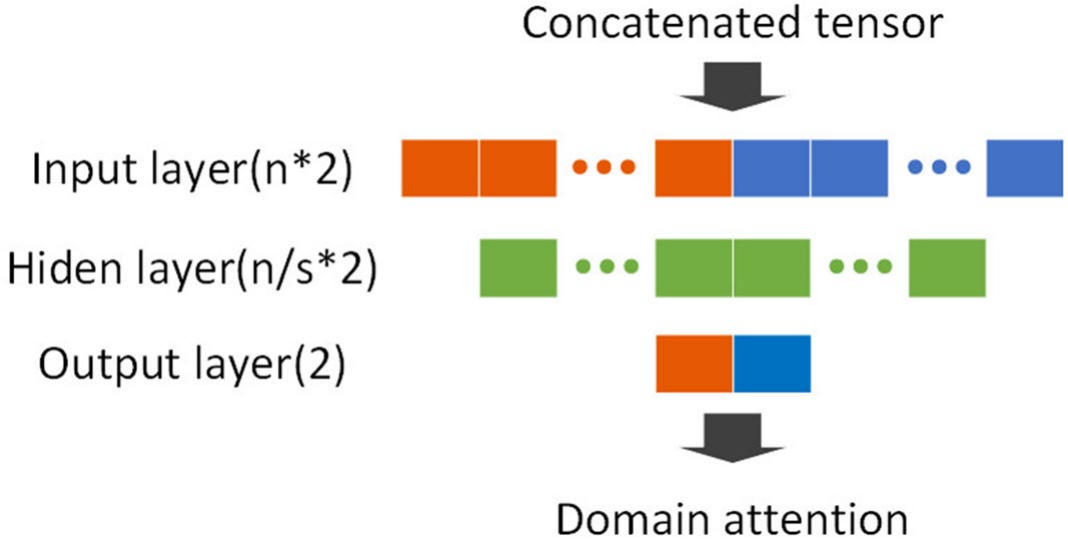
Flowchart of the domain attention unit. s is the compression ratio.

The structure of domain attention module is shown in Fig. 3. It outputs a domain-sensitive weight tensor (domain attention) that is used to recalibrate the raw channel-wise attention obtained from the two SE units. The domain attention vector is generated by the following formula:

2 $$\begin{aligned} \begin{aligned} X_{DA} = softmax(FC_2(Relu(FC_1(X_{raw})))), \end{aligned} \end{aligned}$$

where $X_{raw}$ is the input of the domain attention unit. $FC_n$s are fully connected layers, *Relu* is a nonlinear activation function. *Softmax* is the normalized exponential function that maps the output of $FC_2$ to a probability distribution.

As shown in Fig. 2, adaptive channel-wise attention units use feature maps in the CNN architecture as their inputs, and their outputs are channel-wise attention tensors. We use the squeeze-and-excitation structure and both global max pooling and global average pooling to generate two kinds of attention tensors. They are concatenated within the channel dimension for subsequent usage. We call the concatenated tensor the ‘raw attention tensor’. Formally, suppose the input of the adaptive channel-wise attention module is *X*; then, the raw attention tensor is generated by the following formula:

1 $$\begin{aligned} \begin{aligned} X_{raw} = Concat(SE_{max}(X)),SE_{avg}(X)), \end{aligned} \end{aligned}$$

where the $SE_{max}$ and $SE_{avg}$ are used to generate the channel-wise attention tensor based on global max pooling and global average pooling, respectively. They are concatenated to generate raw attention tensors of dimension $2c*1$.

Then, we use the domain attention and raw attention tensor to generate the adaptive channel-wise attention:

3 $$\begin{aligned} \begin{aligned} X_{weighted} = Scale(X_{raw}, X_{DA}), \end{aligned} \end{aligned}$$

where $X_{weighted}$ is the adaptive channel attention. *Scale* is matrix multiplication operation used to weight the raw attention tensor $X_{raw}$ by the domain attention $X_{DA}$.

### Spatial attention

Different layers of the CNN contain spatial features of objects of different dimensions. Low layers of the CNN mainly contain edge and corner features. As the layers deepen, they contain higher-dimensional features of objects, such as features of objects parts or the whole objects. As a result, it is important to focus the computational resources on the most informative positions with respect to spatial dimensions. Generally, channel-wise attention resolves what to focus on; spatial attention resolves where to focus on. We designed a spatial-wise attention module to improve the performance of YOLOv3. The proposed spatial attention module is also lightweight and fully data driven.

**Figure 4:**
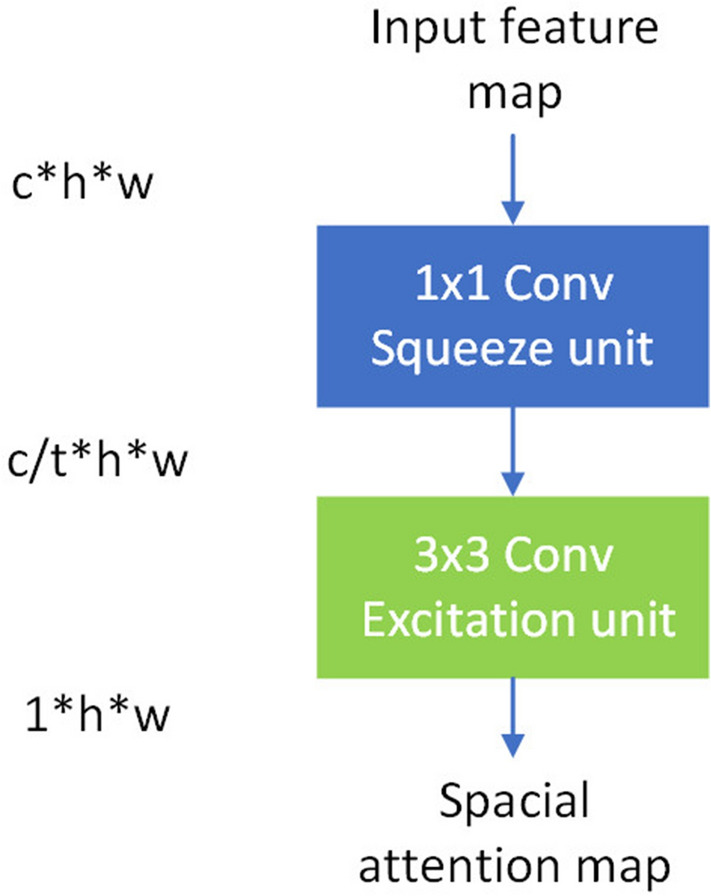
Flowchart of the spatial attention unit. t is the compression ratio.

Different from^9^, which generated spatial-wise attention though both global max pooling and global average pooling across the channel dimension, we generate spatial-wise attention through fully convolutional layers in a learning manner. The proposed spatial attention module is shown in Fig. 4. It produces a spatial attention map to recalibrate the features in different spatial locations. Because the spatial attention unit is composed of a $1\times 1$ convolution layer and a $3\times 3$ convolution layer, the relative position and receptive field of pixels on the spatial attention map are the same as the output of the backbone layers. As a result, the pixels on spatial attention map only weight the pixels of the same locations of the output feature maps. The $1\times 1$ convolution layers are used to squeeze the feature map across the channel dimension. It also prevents the direct influence of backpropagation on the backbone network. The $3\times 3$ convolution layers are used to excite a local area response to amplify their efficiency. The spatial-wise attention is generated by the following formula:

4 $$\begin{aligned} \begin{aligned} X_{sp} = f_3(f_1(X)) \end{aligned} \end{aligned}$$

where $f_1$ and $f_3$ are the $1\times 1$ and $3\times 3$ convolution layers with nonlinear functions, respectively.

### Integrating the attention modules with YOLOv3

YOLOv3 integrated many advantageous design concepts of the CNN such as residual connections, $1\times 1$ convolution kernels, and detectors with multi-scale features. We improve the performance of YOLOv3 by integrating YOLOv3 with the adaptive channel-wise attention, domain attention and spatial-wise attention proposed in the previous two subsections.

The proposed attention modules are easily implemented in a plug-in manner. We only apply spatial attention to lower layers of several modules of YOLOv3 as the spatial dimension of higher layers is small; thus, they contain little positional information. On the other hand, we only apply channel attention to higher layers as the channel dimension of lower layers is also small; thus, they contain little semantic information. Furthermore, modern CNN-based detectors rely largely on transport learning. We do not modify the first few layers so that we can make use of pre-trained DarkNet53 model to initialize the first few layers of the proposed network during the training stage.

**Figure 5:**
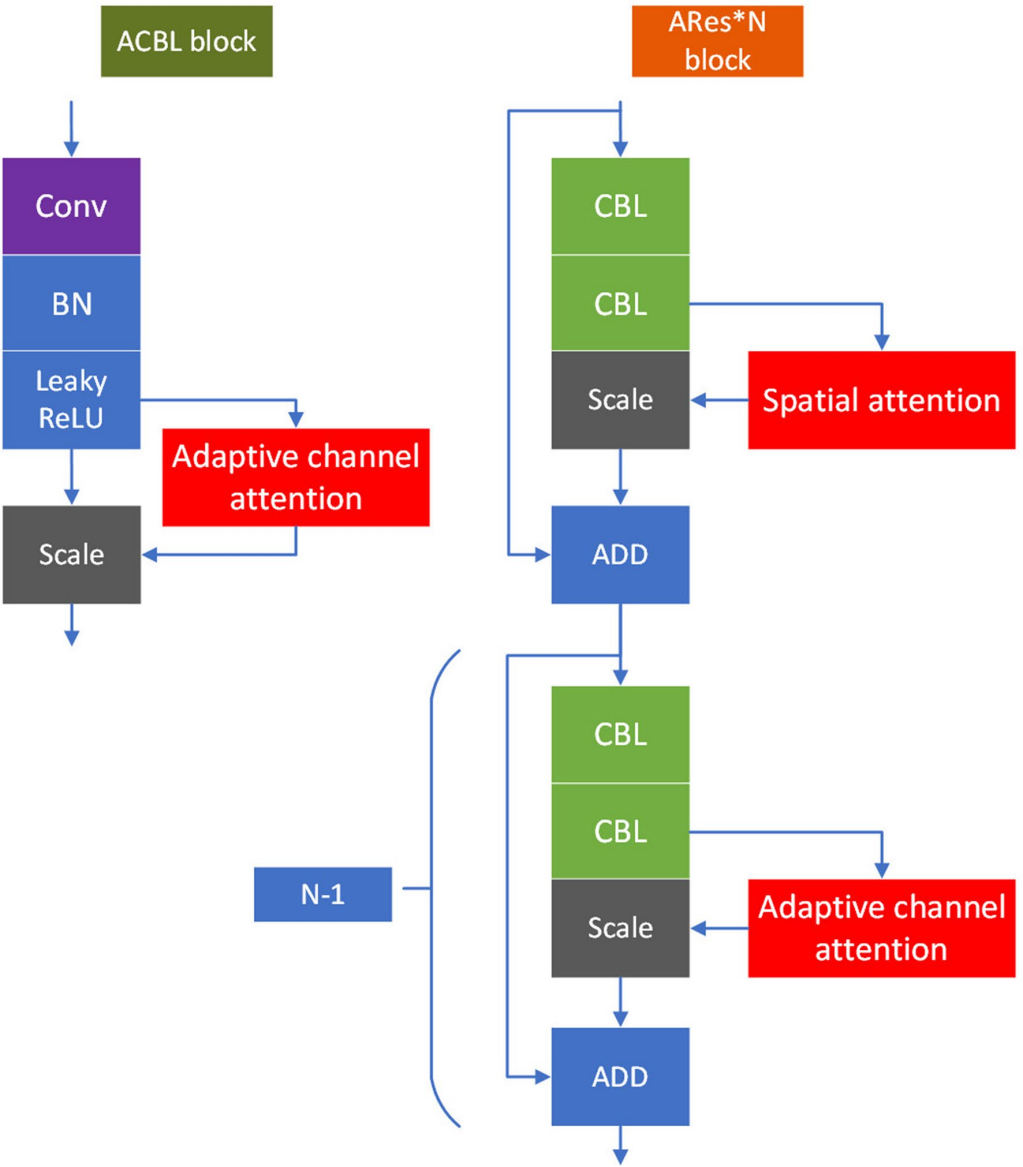
Detailed intro-block connections of ‘ACBL’ and ‘ARes*N’.

According to the above analysis, we designed a novel network based on the YOLOv3 model. It is shown in Fig. 1. As shown in the figure, adaptive channel-wise attention modules reside in both ‘ACBL’ modules and ‘ARes*N’ blocks. On the other hand, spatial-wise attention modules reside only in ‘ARes*N’ blocks. The detailed intro-block connections of ‘ACBL’ and ‘ARes*N’ are shown in Fig.  5. In the next section, we will introduce how to train and evaluate the proposed model in detail.

## Experimental results and analysis

The performance of the proposed improved YOLOv3 model was evaluated on the KITTI dataset^25^ and PASCAL VOC 2007 dataset^26^. We carried out experiments with single NVIDIA 1080 Ti GPU with 8 GB of main memory and a Core i7 processor (3.2 GHz) on the Ubuntu 16.04 operating system. The code was implemented in TensorFlow and compiled with cuDNN. The compression ratios r, s, and t, respectively, in Figs. 2, 3 and  4 are set to 16, 16 and 32, in both experiments on the KITTI and Pascal VOC datasets.

### Experiment on KITTI dataset

The KITTI object detection dataset contains 7381 training images with ground truth labels. It contains 3 categories of objects: vehicles, people and cyclists. The objects to be detected are of various sizes and contain many small samples which make it a challenging task. The labels of the KITTI testing set are not publicly available, so we split the training images in half into a training set and a validation set. In all the experiments, the training dataset was used for training, and the validation dataset was used for testing. For a fair comparison, we used the same training set and validation set for all experiments.

For the experiment on the KITTI dataset, the training is divided into two stages. In the first stage, the backbone network is frozen, and the weights of the network are only updated after the conv 52 layer. In the second stage, whole network is updated. The Adam optimizer with a default learning rate of 0.001 at the beginning is used and is re-initialised after the first stage is finished. Both the first stage and the second stage are trained for 40 epochs. We scale the input image size into various sizes, such as $320\times 320$ and $352\times 352$, in the training stage. In the evaluation stage, the input image size is scaled into 544 $\times$ 544 to achieve a better performance. We randomly split the 7381 training images in half into a training set and a validation set. The mean average precision (mAP) results are evaluated on the validation set. The batch size is 6. The weights of backbone layers of the model are initialized by the DarkNet53 model pre-trained on ImageNet. We stop training after the 200th epoch. Data augmentation techniques such as random cropping and flipping are adopted to avoid overfitting. The model was pre-trained on the COCO dataset^27^ and fine-tuned on the Pascal VOC dataset.

The performances of the models are evaluated based on mAP, inference time, model size and Gflops. We re-evaluated YOLOv3 and YOLOv3 with SE units for a fair comparison. Other studies are trained using the default settings in the official code of each algorithm. We compared the performance of our proposed model with the original YOLOv3, YOLOv3 with SE modules, and our proposed model. To test the effect of each part, we conduct a control experiment. First, we test the original YOLOv3 model on the KITTI dataset. Second, we use only the global average pooling scheme or only the global average pooling strategy to test the effects of each branch. Third, we test the two-branch structure. We do not use adaptive domain attention in this experiment. This is a special case of adaptive domain attention that equivalently weights the output of each branch of the two-branch structure. Fourth, we test YOLOv3 with the proposed adaptive channel-wise attention module. Last, we test YOLOv3 with both the adaptive channel-wise attention module and adaptive spatial-wise attention module. The performance for each configuration is shown in Table 1.

**Table 1:** Comparison of the proposed model with recent works on KITTI dataset.

Method	Car	Pedestrian	Cyclist	mAP	Inference time (ms)	Model size (M)	Gflops
YOLOv3 (original)	91.1	74.6	79.0	81.6	47	246.3	112.6
YOLOv3+SE (GMP)	91.4	75.1	81.6	82.7	49	251.6	115.3
YOLOv3+SE (GAP)	91.2	75.5	82.2	83.0	49	251.6	115.3
YOLOv3+SE (GAP and GMP)	91.7	75.6	82.8	83.4	50	257.0	117.2
YOLOv3+ACA (ours)	92.3	77.0	83.4	84.2	50	262.3	119.9
YOLOv3+ACA+ASA (ours)	92.6	77.4	83.6	84.5	51	262.5	120.0

As shown in the table, both branch structures and the adaptive channel-wise attention have positive effects on the original YOLOv3 model, and the proposed adaptive attention module achieves better performance than the other methods in the table. The proposed model improves the mAP value by 2.9%, while YOLOv3 with the SE unit improves the mAP value by 1.4%. The proposed model outperforms the other ones with a small increase in inference time. Besides, we compare the number of trainable parameters and GFLOPs of the models in Table 2. GFLOPs for each model are measured with input images of size $544\times 544$. From the table, the proposed model achieves better performance with a small model size and computational complexity increase. We believe the performance improvement is mainly due to the innovative architecture.

We compare the proposed model with recent works (Gaussian YOLOv3^28^, RefineDet^29^, RFBNet^30^) and a two-stage detection model (MS-CNN)^31^ in Table 2.

**Table 2:** Evaluation results on KITTI dataset.

Method	Car	Pedestrian	Cyclist	mAP	Inference time (ms)
Gaussian YOLOv3^28^	87.3	79.9	83.6	83.6	47
RefineDet^29^	92.7	78.5	83.6	81.9	72
SSD^19^	85.1	48.1	50.7	61.3	69
RFBNet^30^	86.4	61.6	71.7	73.4	51
SqueezeDet+^32^	85.5	73.7	82.0	80.4	31
MS-CNN^31^	87.4	80.4	86.3	84.7	246
YOLOv3+adaptive attention (ours)	91.2	75.0	81.3	84.5	51

As shown in the table, the proposed model achieves better performance than most of the other models in the table. MS-CNN achieves a similar performance to the proposed model, but it runs much slower than the proposed model.

### Experiment on the Pascal VOC dataset

**Figure 6:**
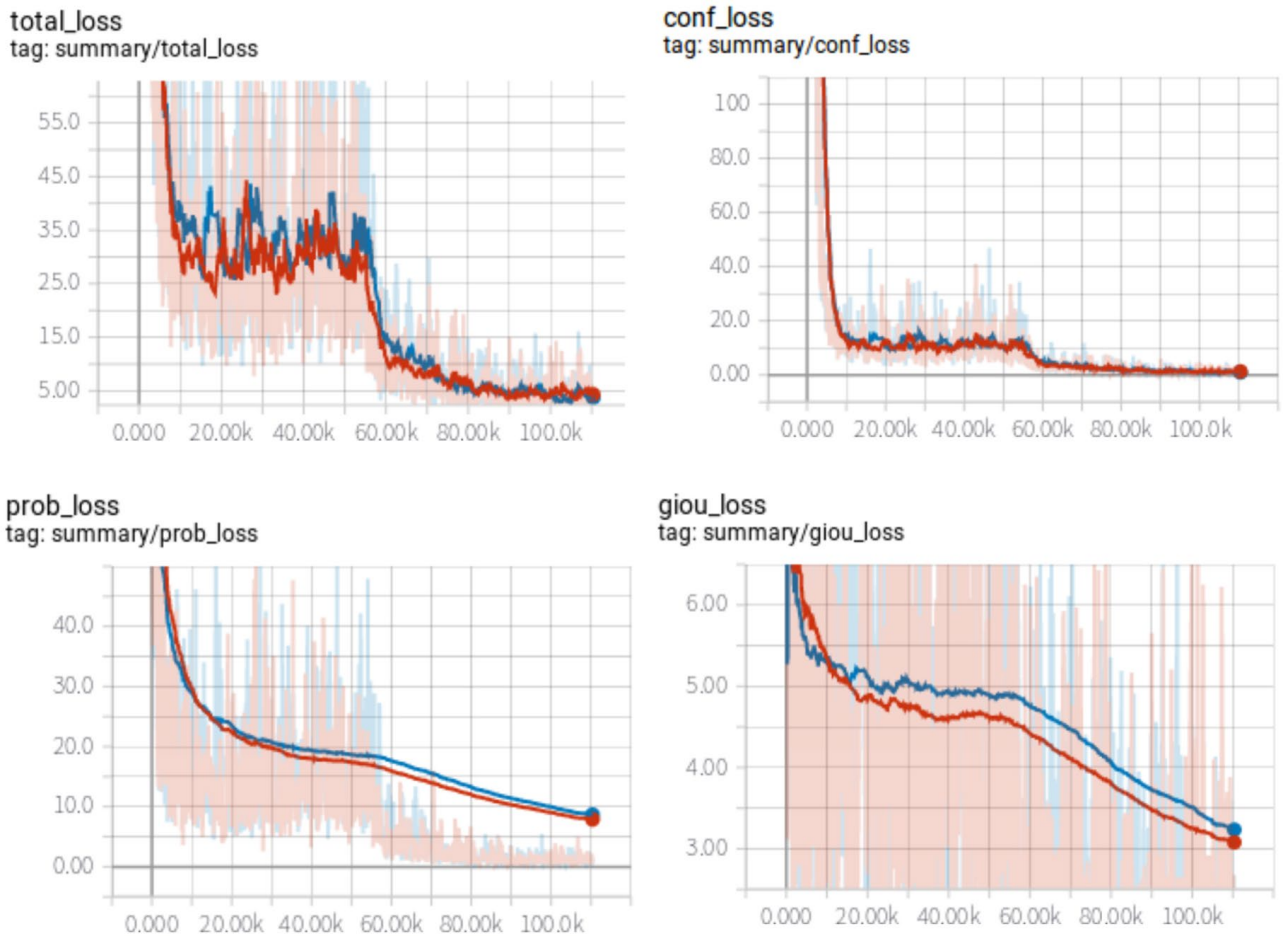
Loss curves.

**Figure 7:**
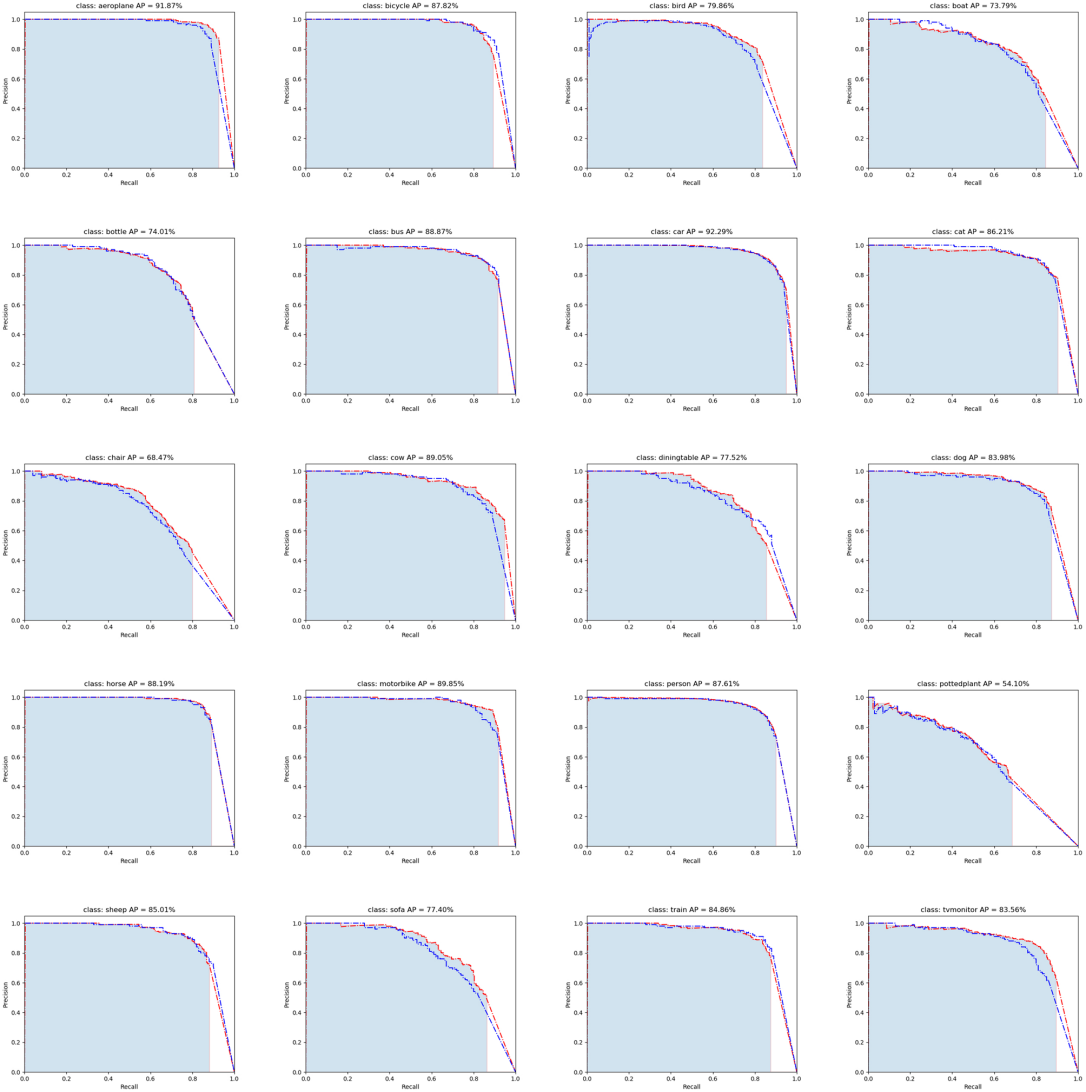
AP curves for each class.

The Pascal VOC dataset contains three computer vision tasks: classification, detection and segmentation. For the object detection task, it has 20 different classes to be detected such as people, birds, and cats. The VOC 07+12 train and val sets are employed for training, and the VOC 07 test set is employed for evaluation. In this experiment, the models were pre-trained on the COCO dataset and fine-tuned on the Pascal VOC dataset, which needs only a few epochs to make the training converge.

The training is divided into two stages. In the first stage, the backbone network is frozen, and the weights of the network are only updated after the conv 52 layer. In the second stage, the whole network is updated. Both the first stage and the second stage are trained for 20 epochs. We scale the input image size into various sizes, such as $320\times 320$ and $352\times 352$, in the training stage. In the evaluation stage, the input image size is scaled into $544\times 544$. The loss curve for each model is shown in Fig. 6. The curves with red colour and blue colour depict the training loss of the proposed model and the original YOLOv3 model, respectively. Figure 6a shows the total loss curve, which is the summation of the other three losses. As shown in Fig. 6b, the red curve and blue curve almost coincide with each other, denoting that the proposed method does not affect the foreground and background classification much. Given a set of anchors with a positive tag, Fig. 6c and d show that the initial $prob\_loss$ and $giou\_loss$ of the proposed model are larger than that of the original YOLOv3. This is because adaptive attention units added new weights to the model. However, as the training goes on, the red curves descend faster than do the blue curves. Thus, the adaptive attention weights towards more informative weights and is the reason that YOLOv3 with the adaptive attention mechanism achieved better performance. For each class, the PR-curves are shown in Fig. 7. As shown in the sub-figures, in most cases, the performance of the original YOLOv3 is worse than that of the proposed model. Finally, we list the mAP and inference time of the original YOLOv3 and the proposed model in Table 3.

**Table 3:** Evaluation results on the PASCAL VOC dataset.

Method	mAP	Inference time (ms)
YOLOv3	81.0	49
YOLOv3+adaptive attention (ours)	82.2	54

As shown in the table, the proposed model with adaptive attention modules achieves a better performance than YOLOv3 with a small increase in inference time. This research sheds new light on the design of attention mechanism modules.

### Experiment on MobileNetv2 with modified SSD detector

**Figure 8:**
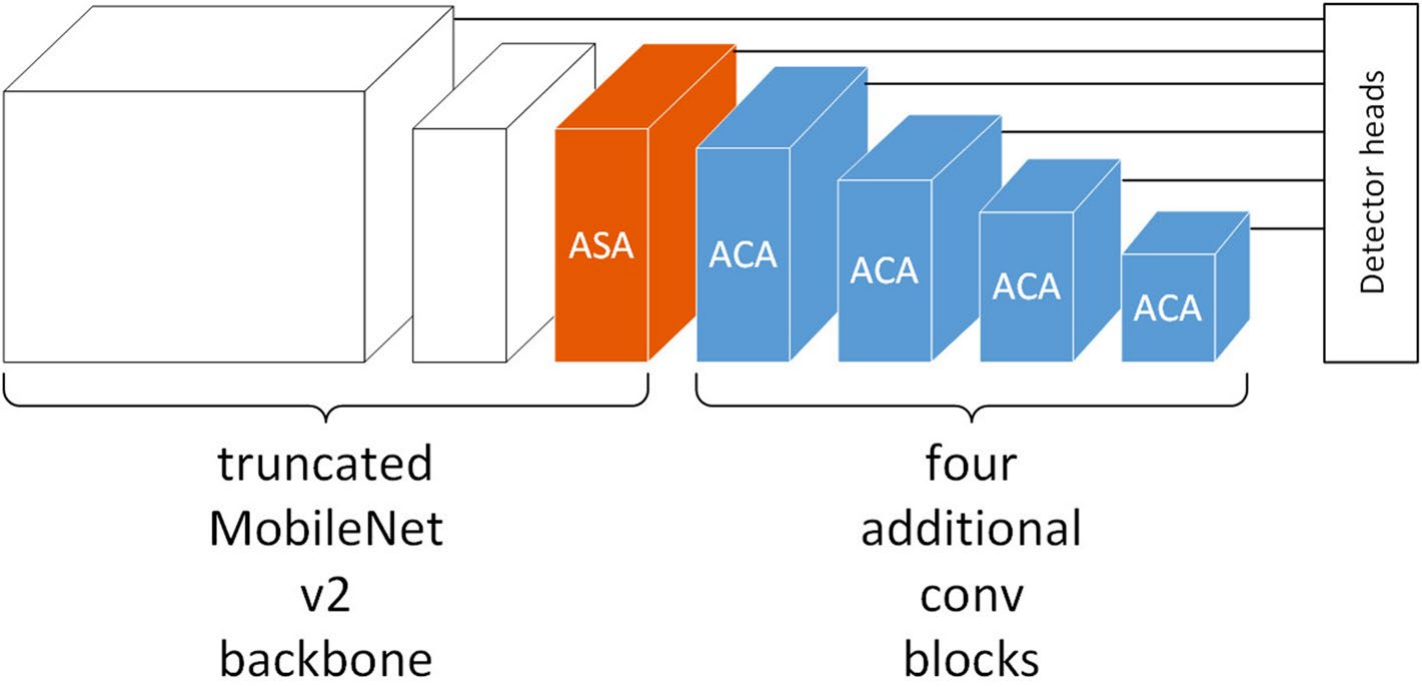
MobileNetv2 with modified SSD detector model. ASA is adaptive spatial-wise attention. ACA is adaptive channel-wise attention.

**Figure 9:**
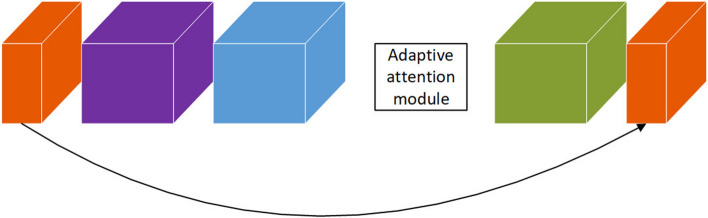
Inverted residual module. ASA or ACA is used to recalibrate the feature maps (blue cube) generated by the point-wise convolution layer within the IRM module.

We evaluate the proposed adaptive attention over MobileNetv2 with a modified SSD detector (Fig. 8). MobileNetv2 is built upon inverted residual modules(IRMs) (Fig. 9). We added four additional convolution blocks behind the truncated MobileNetv2 backbone network. Each of the four additional convolution blocks is built upon two sequential IRMs. The modified SSD detector heads are connected behind the IRM5_3, the last IRM of the truncated MobileNet, and each of the four additional convolution blocks. There are six detector heads in total. We apply the adaptive spatial-wise attention module in the last IRM of the truncated MobileNetv2 backbone that has large feature maps in the spatial dimension (orange cube in Fig. 8), and apply the adaptive channel-wise attention module to the following four IRMs that have more feature map channels (blue cube in Fig. 8). In the experiment, the adaptive attention module is used to recalibrate the feature maps generated by the point-wise convolution layer within the IRM module (Fig. 9).

We test the original model and the model with the adaptive attention modules on the PASCAL VOC dataset. The experimental results are shown in Table 4. We use mAP to evaluate both models. As shown in the table, the mAP value of the proposed model is 1.7 times higher than the original model.

**Table 4:** MobileNetv2 with modified SSD detector evaluation results on the PASCAL VOC dataset.

Method	mAP	Inference time (ms)
MobileNetv2 with modified SSD detector	68.4	3.66
MobileNetv2 with modified SSD detector + adaptive attention	70.1	3.92

## Conclusion

In this paper, a novel adaptive attention mechanism was proposed to build up attention units that are fully date driven. Based on this principle, three kinds of attention units, namely, an adaptive channel-wise attention unit, an adaptive domain attention unit and an adaptive spatial-wise attention unit, were proposed. They were both lightweight and easy to apply. We applied these adaptive attention units to the YOLOv3 and MobileNetv2 architecture in a plug-in manner. The proposed model was evaluated on the KITTI and Pascal VOC datasets. The experimental results show that the performance was improved with a small increase in inference time compared with the original YOLOv3 and MobileNetv2 architecture. We believe the performance improvements are mainly due to the innovative architecture. Thus, the issues mentioned in the introduction section were resolved.

In the future, our challenge is to apply the proposed method to other computer vision tasks, such as semantic segmentation, and serve this function better.
